# A comprehensive epidemiological approach documenting an outbreak of H5N1 highly pathogenic avian influenza virus clade 2.3.4.4b among gulls, terns, and harbor seals in the Northeastern Pacific

**DOI:** 10.3389/fvets.2024.1483922

**Published:** 2024-11-01

**Authors:** Katherine H. Haman, Scott F. Pearson, Justin Brown, Lauren A. Frisbie, Sara Penhallegon, Azeza M. Falghoush, Rebecca M. Wolking, Brandi K. Torrevillas, Kyle R. Taylor, Kevin R. Snekvik, Sarah A. Tanedo, Ilai N. Keren, Elizabeth A. Ashley, Casey T. Clark, Dyanna M. Lambourn, Chrissy D. Eckstrand, Steven E. Edmonds, Emma R. Rovani-Rhoades, Hanna Oltean, Kristin Wilkinson, Deborah Fauquier, Allison Black, Thomas B. Waltzek

**Affiliations:** ^1^Wildlife Program, Science Division, Washington Department of Fish and Wildlife, Olympia, WA, United States; ^2^Department of Veterinary and Biomedical Sciences, Pennsylvania State University, University Park, PA, United States; ^3^Washington State Department of Health, Shoreline, WA, United States; ^4^Center Valley Animal Rescue, Quilcene, WA, United States; ^5^Washington Animal Disease Diagnostic Laboratory, Pullman, WA, United States; ^6^Department of Veterinary Microbiology and Pathology, Washington State University College of Veterinary Medicine, Pullman, WA, United States; ^7^EpiCenter for Disease Dynamics, One Health Institute, School of Veterinary Medicine, University of California Davis, Davis, CA, United States; ^8^West Coast Regional Office, National Marine Fisheries Service, Seattle, WA, United States; ^9^Office of Protected Resources, National Marine Fisheries Service, Silver Spring, MD, United States

**Keywords:** gull, highly pathogenic avian influenza (HPAI H5N1), H5N1 2.3.4.4b, marine mammals, harbor seal, tern

## Abstract

Highly pathogenic avian influenza viruses (HPAIV) H5N1 clade 2.3.4.4b continue to have unprecedented global impacts on wild birds and mammals, with especially significant mortality observed in colonial surface-nesting seabirds and in some marine mammal species. In July of 2023 H5N1 HPAIV 2.3.4.4b was detected in Caspian terns nesting on Rat Island, Washington USA. An estimated 1,800–1,900 adult terns populated the breeding colony, based on aerial photographs taken at the start of the outbreak. On a near-weekly basis throughout July and August, we counted and removed carcasses, euthanized moribund birds, and collected swab and tissue samples for diagnostic testing and next-generation sequencing. We directly counted 1,101 dead Caspian tern adults and 520 dead chicks, indicating a minimum 56% loss of the adult colony population and potential impacts to reproductive success. Combining the observed mortality on Rat Island with HPAI-related Caspian tern deaths recorded elsewhere in Washington and Oregon, we estimate that 10–14% of the Pacific Flyway population was lost in the summer of 2023. Comparatively few adult Glaucous-winged gulls (hybrids) nesting on Rat Island died (~3% of the local population), although gull chick mortality was high. Sixteen harbor seals in the immediate or nearby area stranded during the outbreak, and H5N1 HPAIV was detected in brain and/or lung tissue of five seals. These cases are the first known detections of HPAIV in a marine mammal on the Pacific coast of North America. Phylogenetic analyses support the occurrence of at least three independent avian-mammalian virus spillover events (tern or gull to harbor seal). Whole genome sequencing indicated that H5N1 HPAIV may have been introduced to Washington from Caspian terns in Oregon. Ongoing monitoring and surveillance for H5N1 HPAIV in the marine environment is necessary to understand the epidemiology of this virus, assess conservation impacts to susceptible species, and provide support for data-driven management and response actions.

## 1 Introduction

Since 2020, H5N1 clade 2.3.4.4b highly pathogenic avian influenza virus (HPAIV) has caused significant impacts to wild birds and mammals globally, with confirmed detections in millions of animals and over 500 species (1). Historically HPAIV were rarely detected in wild birds, but rather emerged in poultry after a wild bird-origin low pathogenic avian influenza virus (LPAIV) was introduced and adapted to a gallinaceous host (2). A paradigm-shift emerged in the early 2000s as the number of H5N1 wild birds became increasingly involved in HPAI epidemiology (3). This shift initially manifested as sporadic wild bird mortalities associated with H5N1 HPAIV and, subsequently, long distance dissemination of the virus by wild birds (2, 3). With the emergence of H5 clade 2.3.4.4b HPAIV, the extent and diversity of hosts, virulence, and transmission and maintenance dynamics associated with HPAIV radically changed (2, 4–6). H5N1 clade 2.3.4.4b HPAIV has caused sporadic deaths and mass die-offs in wildlife across Europe, Africa, Asia, North America, and South America since 2020 (7–12). In addition to wild birds, thousands of domestic and wild mammals have died because of spillover events and, in certain cases, likely mammal-mammal transmission (13–16). Thus, while HPAI has always posed a threat to domestic animal health, commercial agriculture, and food security, its risk to public health and wildlife conservation has drastically intensified.

Despite the apparent widespread distribution and continued circulation of H5N1 HPAIV in wildlife, disease presentations and outcomes vary among avian and mammalian hosts. The pathobiology of HPAIV is complex and involves the interaction of multiple factors related to level of exposure, viral strain, and host (17). A wide range of clinical responses to H5N1 HPAIV have been documented in taxonomically similar species, even under controlled experimental conditions (18–23). Interspecific variation in susceptibility to infection and disease is magnified by differences in the biology and behaviors of different species that may impact viral exposure, as well as any pre-existing immunity from prior low pathogenic avian influenza (LPAI) virus infections (3, 19, 24, 25). Regarding avian-origin influenza A viruses (IAVs), gulls and terns (Family Laridae) are frequently discussed as a collective group. However, while gulls are well-studied hosts of LPAI and HPAI viruses, there is a dearth of information on IAV pathobiology and ecology in terns, and field observations during the ongoing H5N1 HPAIV panzootic suggest important differences may exist between the two avian groups.

Gulls are recognized reservoirs for LPAIV and can be infected with a wide-diversity of subtypes, including the gull-adapted H13 and H16 subtypes (26, 27). Gulls are clinically susceptible to H5N1 HPAIV (18, 19); however, some field evidence suggests lower mortality rates in adult gulls relative to juveniles, presumably associated with preexisting immunity. In contrast to gulls, large scale mortality events in terns associated with H5N1 HPAIV have been evident since 2020. For example, Sandwich terns (*Thalasseus sandvicensis*) were susceptible to H5N1 HPAIV, with extremely high mortality observed in both adults and juveniles on nesting colonies in the Netherlands (12). Mortality in Sandwich terns across Northwestern Europe is thought to have resulted in a 17% loss of the global breeding population (28). In the United States, there were reports of high mortality rates in Caspian terns (*Hydroprogne caspia*) in the Great Lakes region of Michigan and Wisconsin (29, 30), though published epidemiological data are limited. The distinct lack of information on IAVs in terns limits our ability to interpret the short and long-term impacts of H5N1 HPAIV, particularly when only mortality data are available.

There are numerous challenges associated with discerning epidemiological patterns of HPAIV outbreaks, especially in free-ranging wildlife when only field observations are available. This is true for both individual mortality events and for events over greater spatial and temporal scales. Pairing on-the-ground outbreak investigation and surveillance efforts with viral molecular epidemiology is crucial to understanding patterns of transmission. It is also of critical importance to combine disease surveillance with population monitoring to provide a denominator for simple mortality counts and assess immediate and long-term implications for wildlife conservation and management.

Adopting a comprehensive approach, we describe an H5N1 HPAIV mass mortality event that affected Caspian terns, Glaucous-winged and Western gull hybrids (*Larus occidentalis x glaucenscens*), and harbor seals (*Phoca vitulina*) on Rat Island, Washington USA in the summer of 2023. This event occurred approximately 16 months after the first detection of H5N1 HPAI virus in a wild bird in Washington and involved the first confirmed detection of HPAIV in a marine mammal in the Northeast Pacific. We combine traditional surveillance and outbreak investigation approaches with population monitoring and molecular epidemiology to describe the mass mortality event and quantify its impact on the Pacific Flyway Caspian tern population.

## 2 Materials and methods

### 2.1 Study area and species

**Figure 1:**
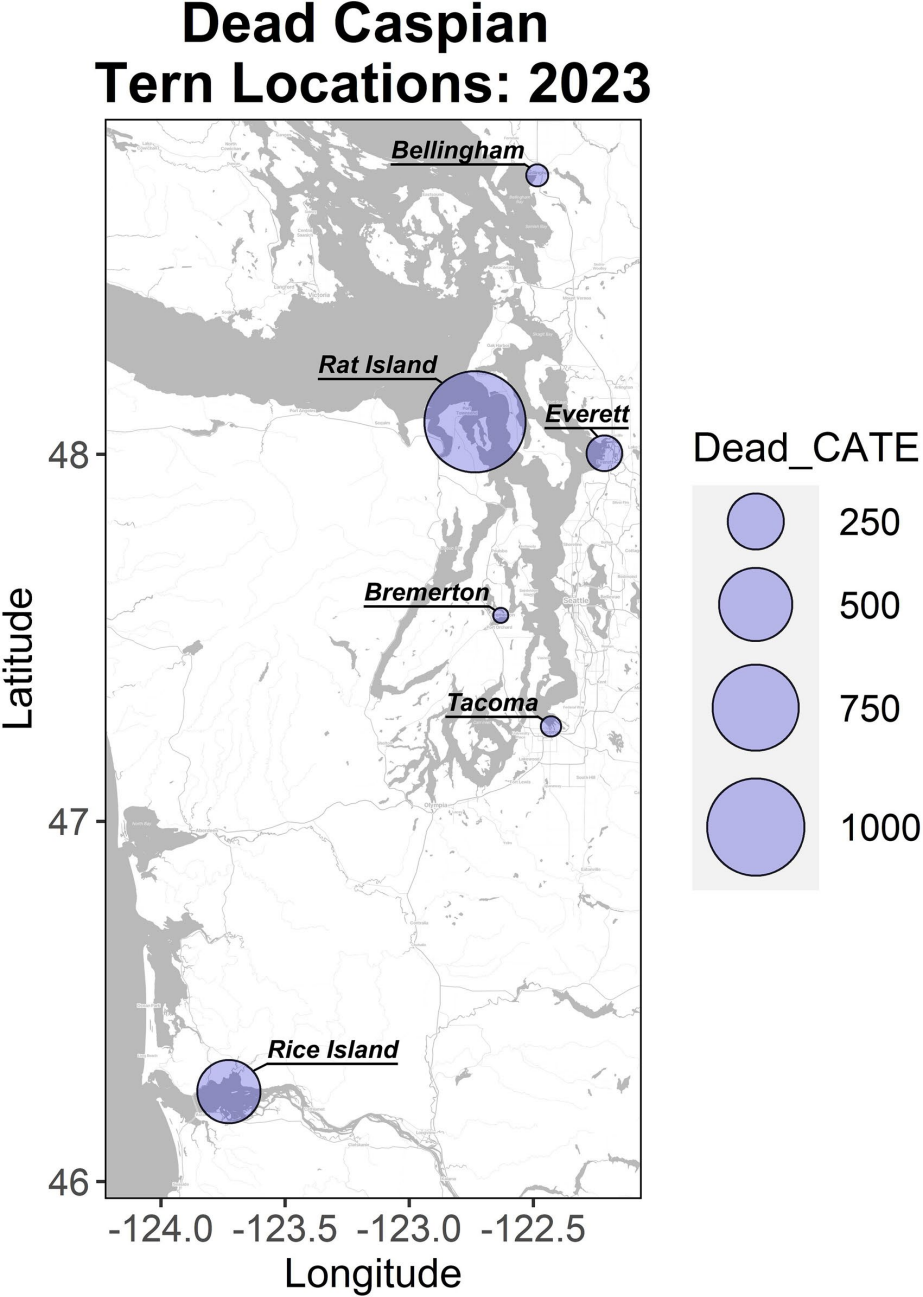
Localities and magnitude of Caspian tern mortality in Washington and Oregon in the summer of 2023. Rat Island incurred the largest mortality and is the focus of this study. The Rice Island location along the lower Columbia River includes birds from East Sand Island and Astoria, which are located downstream of this location.

This study occurred primarily on Rat Island in the Salish Sea, but also includes data on Caspian tern mortality in other localities in the Salish Sea and on the lower Columbia River estuary of Oregon and Washington (Figure 1). The Salish Sea is a 16,925 km^2^ inland sea extending from Olympia, Washington, USA, north to Campbell River, British Columbia, Canada, and includes Puget Sound, the Strait of Georgia, and the Strait of Juan de Fuca. Rat Island is in Puget Sound’s Port Townsend Bay. It is a low-lying and sparsely vegetated island (primarily by grasses and herbaceous flowering plants) that is approximately 4.26 ha in size and 0.61 km at its longest axis. The island is used by migrating and over-wintering waterfowl and shorebirds and by several species of breeding birds and one mammal including, Caspian terns, Glaucous-winged and Western gull hybrids (hereafter gulls), black oystercatchers (*Haematopus bachmani*), savannah sparrows (*Passerculus sandwichensis*), song sparrows (*Melospiza melodia*), and harbor seals. Only gulls, terns and seals were observed dead or moribund on Rat Island during carcass collection trips (described below).

Caspian terns are migratory and populations on the U.S. West Coast spend their winters primarily in southern California and Mexico and return to the Salish Sea in late April and early May. They breed in large and dense colonies where they form depressions in the soil and lay 2–3 eggs. Gulls are found on Rat Island year-round and lay 2–3 egg clutches annually in June. On Rat Island in 2023, tern eggs started hatching in early July and continued to hatch through August. Harbor seals use Rat Island year-round as a haulout and pupping site, or rookery. The number of harbor seals hauled out at any given time ranges from zero to 234 non-pups (adults, subadults, and weaned pups) and zero to 54 pups, depending on the year, day, and tide conditions [raw counts are published in (31)]. Harbor seal pupping occurred in late June and continued through early August of 2023, which is typical of most years. Prior to the outbreak on Rat Island, H5N1 HPAI virus had been circulating in Washington State since March of 2022. The virus was primarily detected in waterfowl, shorebirds and predatory and scavenging bird and mammal species and was not associated with this marine ecosystem.

### 2.2 General surveillance and disease outbreak investigation

The first reports of sick or dead Caspian terns were made to the Washington Department of Fish and Wildlife on July 10, 2023. Given the proximity of Rat Island to Fort Flagler State Park, a very populated state park and campground, we began visiting the island to remove carcasses on July 17, 2023. During this active outbreak, we conducted near weekly site visits for sample collection for pathogen surveillance and carcass counting and removal through August 11, 2023. We also conducted two additional site visits after what we considered to be the active outbreak period, in mid-September and early October (Supplementary Tables S1, S2).

### 2.3 Sample collection

Over the course of the outbreak select carcasses were sampled for IAV testing. Briefly, for wild avian species, pooled choanal / cloacal samples collected with sterile polyester tipped plastic handle swabs (Puritan, Maine USA^®^) were collected from individual birds and placed in viral transport media (BD Universal Viral Transport for Viral, Chlamydial, Mycoplasmal, and Ureaplasmal Specimens, New Jersey USA). Swab samples were stored on ice packs in the field and shipped on ice packs to the Washington Animal Disease Diagnostic Laboratory (WADDL) within 48 h of collection. If shipment could not occur within 48 h, samples were frozen at −20C until shipped. Wild bird carcasses were not submitted for postmortem examination due to the ongoing H5N1 HPAIV outbreak in Washington and the known impacts on wild birds.

Harbor seals that were found dead, either by WDFW staff or reported to the local Marine Mammal Stranding Network in the immediate geographic area (within approximately 2 km) of Rat Island, were initially sampled by collecting a nasopharyngeal and a rectal swab in separate vials of transport media. Similar to the avian samples, these were maintained on ice packs in the field and submitted to WADDL within 48 h of being collected, or frozen if shipped after 48 h. However, after several harbor seals stranded dead in the immediate area, we included additional tissue samples for diagnostic testing. If the carcass was small enough to ship (e.g., pup), the whole carcass was submitted to WADDL. From larger individuals (e.g., adults) we sampled lung and the whole head for submission to WADDL for IAV matrix PCR, histology, and immunohistochemistry. All samples were shipped frozen.

### 2.4 Movement patterns of Caspian terns

We recovered federal bands from the legs of dead Caspian terns. The bands were removed, and the unique identification numbers on the bands were sent to the researchers responsible for banding the terns and tracking their resights. Banding data included the original banding location, age of banding (adult or chick), and summaries of re-sights (location and year). This information was used to compare the movement patterns of terns prior to the disease outbreak with the pattern of disease spread observed in Washington and Oregon and to examine potential long-distance movement of H5N1 HPAIV by Caspian terns.

### 2.5 Population estimates

To estimate the number of nesting terns and gulls on Rat Island, oblique aerial photographs were taken on July 17, 2024 during four passes over the island from an airplane flying at ~244-meter altitude and travelling at 148–167 km/h using a digital single lens reflex camera with a 100–400 mm lens with camera speed and International Organization for Standardization (ISO) adjusted to maximize resolution. The photographs included four series of overlapping photos from each pass over the Island that were stitched together in Adobe Photoshop to create a single complete view of the colony from each overflight (overlapping areas were removed to avoid double counting using landmarks) and the following variables were counted from each of the four series of photographs: Caspian tern = live adults, dead adults, nests; gulls = live adults; harbor seals = pups, non-pups (Supplementary Figure S1). Gull nests were difficult to identify from aerial counts because many were obscured by vegetation or driftwood and no dead adult gulls were identified in the photographs. Many dead terns collected during carcass collections were in an incubating position and would have been impossible to distinguish from live, incubating birds from aerial counts. As a result, we did not use the photographs to estimate tern mortality. We combined counts of live and dead adult terns to estimate the total number of adult terns on the colony just prior to the first carcass collection, which served as our estimate of the total number of Caspian terns associated with the Rat Island breeding colony. Each nest or animal was counted in Adobe Photoshop using the count tool to avoid double counting and to help us enumerate all variables (e.g., nests, adults) given the close proximity of nests and birds in the colony.

### 2.6 Documenting mortality and estimating mortality rates

During the active H5N1 HPAIV outbreak we collected all carcasses from Rat Island in four primary sweeps (8–11 days apart) on July 17, July 25, August 2, and August 11, 2023. We conducted two additional sweeps later in the reproductive season (September 13 and October 3, 2023) and consider these dates past the active outbreak window (Supplementary Table S2). The purpose of the delay was to avoid disturbing fledgling Caspian terns. During sweeps, all gull and tern carcasses were counted (adults and chicks), bagged, and removed from the island. All personnel wore hooded Tyvek suits, rubber boots, gloves, goggles, and N95 masks. All non-disposable equipment, including inflatables and life jackets, was disinfected with a 10% household bleach solution immediately after each visit to the island.

Observed mortality data included six carcass collection dates where we conducted total counts of chick and adult Caspian tern carcasses and chick and adult Glaucous-winged gull carcasses. These counts are either culminations of daily mortalities that occurred between collection periods (known periods of time) or, in the case of the first collection (July 17, 2023), an unknown period of daily mortalities.

To describe daily mortality rates as a continuous exponential curve over unobserved times, we used JAGS 4.3.1 (32) to construct a nonhomogeneous Poisson state-space MCMC model. Thus, each observed count $Y_{i}$ , is a random variable from a Poisson distribution with rate $R_{i}$


$$Y_{i}\sim \mathrm{Pois}\left(R_{i}\right)\;i\in \left\{1,2,3,4,5\right\}$$

where $R_{i}=\sum M_{t}I\left(t\left(i-1\right)\leq t\leq t\left(i+1\right)\right)$ is the cumulation of continuous mortality ( $M_{t})$ between collection dates. We placed a 7-to-21-day uniform prior around time 0, the date of the first carcass collection. Although this is an informative prior, it could be considered diffuse given the observed rate of HPAI growth in poultry (33, 34). We wanted to limit this initial growth period because the colony was under daily observation when the first few carcasses were observed on 10 July 2023, 7 days prior to the first carcass collection. Because the colony was being observed daily by both kayakers watching the island as part of natural history tours and by education volunteers with scopes pointed at the colony, we believe the initial observations of carcasses at the edge of the colony were early in the outbreak. At the same time, because the observers could not see into the middle of the colony, it is probable that the disease started to spread within the colony prior to its first detection (hence the 7–21 days). Published research that estimated secondary cases caused by the infection from one individual (R_0_) suggests initial exponential spread and that this spread continues for a few weeks and is faster than the rate of decrease (33, 34). Although these results are for poultry farms, the Caspian tern nest density in the wild [up to 1.48 nests/m^2^; (35)] is not unlike bird densities observed in poultry farms. Therefore, rather than fitting a symmetric logistic growth curve we modeled the likelihood of the latent continuous mortalities as an asymmetric sigmoid:

$M_{t}=\psi (e^{-t/\sigma^{2}}-e^{-t/\phi }$ ).

where $\phi <\sigma^{2}$ are mortality rates before and after the inflection point and ψ is a scaling factor for the asymptote.

We placed fully diffused priors on all hyper parameters. However, while the prior on ψ was the improper Gamma (0,0) distribution, the prior for $\sigma^{2}$ was restricted as uniform Beta (1,1) inverse ratio of ϕ . Similarly, we placed a minimum precision half normal prior on ϕ but centered it around the natural log of the $\frac{y_{1}}{t_{0}}$ suggesting the inflection point likely occurred during around the time of first collection. For consistency, we fit the same model for the Caspian tern chicks as the adults, except we did not need to estimate $t_{0}$ because chick mortality started about the same date as the first carcass collection (July 17, 2023).

### 2.7 Histology and immunohistochemistry

No samples from wild birds were collected for histology. Whole body cadaver or formalin-fixed tissues were submitted from harbor seals found freshly dead on Rat Island, Ft Flagler, or Indian Island (Supplementary Table S1). Tissues were fixed in 10% neutral-buffered formalin and sections embedded in paraffin for standard H&E slide preparation and examination by veterinary anatomic pathologists. Immunohistochemistry was performed on select sections of brain from two harbor seal cases using the Ultra View Red Detection Kit (Roche Indiana, USA) on the Discovery Ultra (Roche Diagnostics, Indiana, USA) automated platform. Before applying the primary antibodies, slides were pretreated with protease 1 enzyme (Roche). Influenza A primary antibody (Meridian Bioscience) was used at a 1:400 dilution in antibody diluent (Roche Indiana, USA) and incubated for 28 min. Slides were briefly counterstained with hematoxylin. Applied antibody diluent without the primary antibody served as the negative control.

### 2.8 Nucleic acid extraction and RT-qPCR for the detection of IAV

Pooled avian choanal/cloacal swabs, nasopharyngeal swabs from harbor seals, and select tissues from harbor seals (lung, brain) were submitted to WADDL for IAV screening with reverse transcription quantitative PCR (RT-qPCR). Nucleic acid extractions of swabs and tissues were performed using a MagMAX™96 Viral RNA Isolation Kit as per the manufacturer’s instructions (Thermo Fisher Scientific, Washington USA). RT-qPCR targeting the IAV matrix gene segment was performed as previously described (36). Samples with evidence of IAVs were submitted to the United States Department of Agriculture (USDA)‘s National Veterinary Services Laboratory for confirmatory testing, subtyping, and pathotyping.

### 2.9 Twist comprehensive viral research panel (CVRP) sequencing and molecular analyses

RNA extracts from non-negative samples (those yielding IAV matrix gene segment RT-qPCR cycle threshold (Ct) values <30) were selected for next-generation sequencing (NGS). The sequencing library was constructed using the Twist Library Preparation EF Kit 2.0 (Twist Bioscience, California USA) followed by viral enrichment by hybrid capture using the Twist CVRP with Standard Hybridization v2 reagents [Twist Bioscience, California USA, (37)]. Hybrid capture was performed according to manufacturer’s instructions. Briefly, a ProtoScript II First Strand cDNA synthesis kit (New England Biolabs, Massachusetts USA) was used to synthesize first strand cDNA, immediately followed by second-strand synthesis using a NEBNext Ultra II Non-Directional RNA Second Strand Synthesis kit (New England Biolabs, Massachusetts USA). After cDNA purification, enzymatic fragmentation, telomere repair, and dA-Tailing, barcoded universal adapters provided by Twist Bioscience were ligated to the cDNA and then purified. The purified barcoded libraires were then PCR-amplified for 15–20 cycles, followed by purification. The DNA concentration of the sequencing libraries was then quantified using a Qubit dsDNA High Sensitivity Assay Kit (Invitrogen, Massachusetts USA) and batches of four to six prepared libraries were pooled by equal mass up to 2 μg total DNA as recommended by Twist Bioscience. Pooled libraries were incubated with hybridization reagents and 1 unit of biotinylated CVRP probes for 15–17 h. Streptavidin beads were then used to purify viral library fragments bound to the CVRP probes. Post-capture PCR was conducted for 12 cycles as recommended by Twist Bioscience to amplify the enriched libraries. The mean fragment length (base pairs) of enriched pools was estimated using a High Sensitivity NGS Fragment Analysis kit (Advanced Analytical, Massachusetts USA) on a Fragment Analyzer capillary electrophoresis system. The concentration of functional, sequenceable molecules in the enriched pools was determined using a KAPA Library Quantification Kit (Roche, Indiana USA). The mean fragment length values and functional concentration values of enriched pools were then used to combine pools to generate the final equimolar library. The final sequencing library was then diluted to 7pM and sequenced on an Illumina MiSeq using a 600-cycle v3 kit.

To generate the consensus genomes, we used QIAGEN CLC Genomics workbench (version 24.0.1). Raw fastq files were imported into CLC Genomics Workbench. Paired-end reads were quality and adapter-trimmed using default parameters (< 2 ambiguities and quality score > 0.05 per read) and aligned to GISAID EpiFlu reference sequence EPI_ISL_18311027. Consensus sequences were called by base majority frequency and no infilling of zero coverage regions, using default parameters. The coding sequences for individual segments comprising the IAV genomes sequenced at WADDL were deposited in GenBank under accession numbers: PQ118625-PQ118632, PQ118638-PQ118645, PQ118399-PQ118406, PQ118380- PQ118387, PQ118169-PQ118176, PQ118050-PQ118057, PQ118039-PQ118046, PQ118001-PQ118008, PQ113434-PQ113441, PQ113408-PQ113415, PQ113396-PQ113403, PQ113381-PQ113388 (Supplementary Table S3).

### 2.10 Dataset curation for phylogenetic analysis

To provide background genomic context for the outbreak sequences, we sourced all hemagglutinin (HA) segment sequences from human and animal hosts, sampled from anywhere in the world, with collection dates between January 1, 2020 through June 5, 2024. Data were downloaded from GISAID.^1^ The metadata for the GISAID sequences in addition to the 12 sequences generated for this study were then assessed for quality and completeness. Briefly, we used seqtk (V1.4) (38) to deduplicate any sequences generated from the same specimen, a process that can occur when originating labs and confirmatory-testing labs both conduct whole genome sequencing. Using custom R scripts, we excluded all sequences generated from samples with egg passage history and any samples with incomplete dates lacking year, month and day of sample collection. Metadata for all samples were then reformatted into the Nextstrain metadata ingest format. This process yielded a dataset of 11,422 cleaned, deduplicated H5N1 HA segments to use as input to phylogenetic analysis in Nextstrain.

### 2.11 Phylogenetic inference using HA gene segments

Using the dataset described above, we performed phylogenetic inference using the Nextstrain software suite (39). This workflow enables subsampling of the data, sequence data alignment, genetic divergence and temporally resolved phylogenetic tree inference, and visualization of the resulting tree using a browser-based package. Briefly, the metadata and fasta files for the 11,422 HA segments were used as the input to Nextstrain Augur. The first step in the pipeline performs subsampling of the genomic data in a tiered way with regard to sampling location. We specified that all sequences sampled from Washington, Oregon, Idaho and British Columbia should be included in the build. Then, sequences sampled from other geographic locations should be sampled at random from the input dataset up to a specified maximum threshold. For sequences sampled from other parts of North America (excluding WA, ID, OR, and BC) we specified inclusion of up to 400 sequences per month per year, and for any geographic location outside of North America, only 2 sequences per month per year should be sampled and included in the build. This subsampling procedure resulted in a dataset of 4,293 HA gene segments used for phylogenetic inference.

As specified within our Nextstrain pipeline, the 4,293 HA segments were aligned with MAFFT (55). The alignment was trimmed to the reference sequence, and a maximum likelihood genetic divergence tree was inferred using IQ-TREE (40). Using the TreeTime package (41), a molecular clock was inferred from the dataset and used to temporally-resolve the tree. The tree structure and associated information were exported as a JSON file for viewing using Nextstrain Auspice. Additional information about the sequences generated for this study were integrated with the genomic analysis using Nextstrain’s metadata overlay feature.

### 2.12 Phylogenetic inference of concatenated whole genome sequences

As a segmented virus, IAV phylogenetic trees are often inferred segment by segment (e.g., one tree for HA, one tree for neuraminidase, etc.). This approach ensures that reassortment events, which can bring together segments with different evolutionary histories, do not confound phylogenetic inference. However, looking at single gene segments yields much shorter sequences, and therefore less visibility into sequence evolution, which can reduce our ability to understand within-outbreak dynamics (42). In cases where reassortment is unlikely, such as geographically limited outbreaks occurring over short timescales, one can concatenate gene segments together to create a whole genome dataset for higher resolution phylogenetic inference.

To perform whole genome phylogenetic inference of the outbreak clade, we concatenated gene segments into a single full-length genome for 17 outbreak-associated viruses that grouped together in a single clade in the HA tree. These 17 sequences included the 12 viruses sequenced at WADDL and five available from GISAID. To ensure proper rooting of this smaller tree, we used an H5N1 concatenated whole genome sampled from a chicken in Oklahoma (*A/chicken/Oklahoma/USDA-013220-001/2022*) as an outgroup. We performed phylogenetic inference as described above, with the addition of reconstructing host species state for internal nodes using TreeTime (41) in Nexstrain. Newick files were visualized with Nextstrain Auspice, and as before we used the Nextstrain metadata overlay feature to integrate additional epidemiological and ecological information with the genomic data.

## 3 Results

### 3.1 General surveillance and disease outbreak investigation

Dead terns and gulls that tested positive for H5N1 HPAIV were first detected on the lower Columbia River in mid-June (2023) and then observed in July and August at multiple locations across the Puget Sound (Washington) including Tacoma, Everett, Bellingham and Rat Island (Figure 1). We focused our intensive investigation of mortality on Rat Island, Washington. The active H5N1 HPAIV outbreak period occurred from early July through the end of August 2023. We visited Rat Island to count and remove carcasses a total of four times during the active H5N1 HPAIV outbreak and two additional times at the end of the outbreak (Supplementary Table S2). During the active outbreak visits, we collected a total of 1,580 Caspian terns, including 1,055 adults, 525 young-of-year, and a total of 211 gulls, including 12 adults and 199 young-of-year. After the active outbreak period, on September 13, 2023, we visited Rat Island and collected 74 terns (30 adults and 44 young-of-year) and 87 gulls (13 adults and 74 young-of-year). At this time, all the Caspian tern carcasses had a prolonged postmortem interval resulting in poor diagnostic quality while 2 adult and 13 young-of-year gull carcasses were considered recently dead (i.e., likely <1 week postmortem). During our final trip to Rat Island on October 3, 2023, we collected 50 young-of-year Caspian terns and 22 young-of-year gull carcasses but found no adult bird carcasses. All the Caspian tern young-of-year carcasses had a prolonged postmortem interval while five of the young-of-year gulls were considered recently dead (Supplementary Table S2). Because of the prolonged postmortem interval in the later carcass collection trips, these birds were not used in generating the mortality curves below. Additional carcasses from these species were collected from Fort Flagler and other areas in Western Washington, as reported to WDFW (Supplementary Table S2).

### 3.2 Sampling

A total of 13 birds (9 terns and 4 gulls) were sampled for diagnostic testing. Of these, six terns (2 young of year and 4 adult) and four gulls (2 young-of-year and 2 adult) were sampled from Rat Island. An additional three terns (all adult) were sampled from surrounding areas (Supplementary Table S1). All nine terns and three of four gulls were IAV-positive via RT-qPCR, and H5N1 clade 2.3.4.4b HPAIV was identified and confirmed in all IAV-positive samples.

A total of 16 harbor seals (6 non-pups and 10 pups) were reported stranded alive (*n* = 1) or dead (*n* = 15) either on Rat Island or nearby Fort Flagler or Indian Island between July 15 and September 10, 2023. All dead stranded harbor seals were in good post-mortem condition except one, that was too heavily scavenged and autolyzed to warrant further investigation (Supplementary Table S1). Dead stranded harbor seals appeared in good body condition with no evidence of gross trauma. Brain, lung, and/or nasopharyngeal swab samples from five harbor seals tested positive for IAV via RT-qPCR; H5N1 clade 2.3.4.4b HPAI virus was identified in all IAV-positive samples. Notably, two harbor seals tested negative for IAV on nasopharyngeal swabs alone but had IAV-positive brain and/or lung tissue samples.

### 3.3 Movement patterns of banded Caspian terns

**Figure 2:**
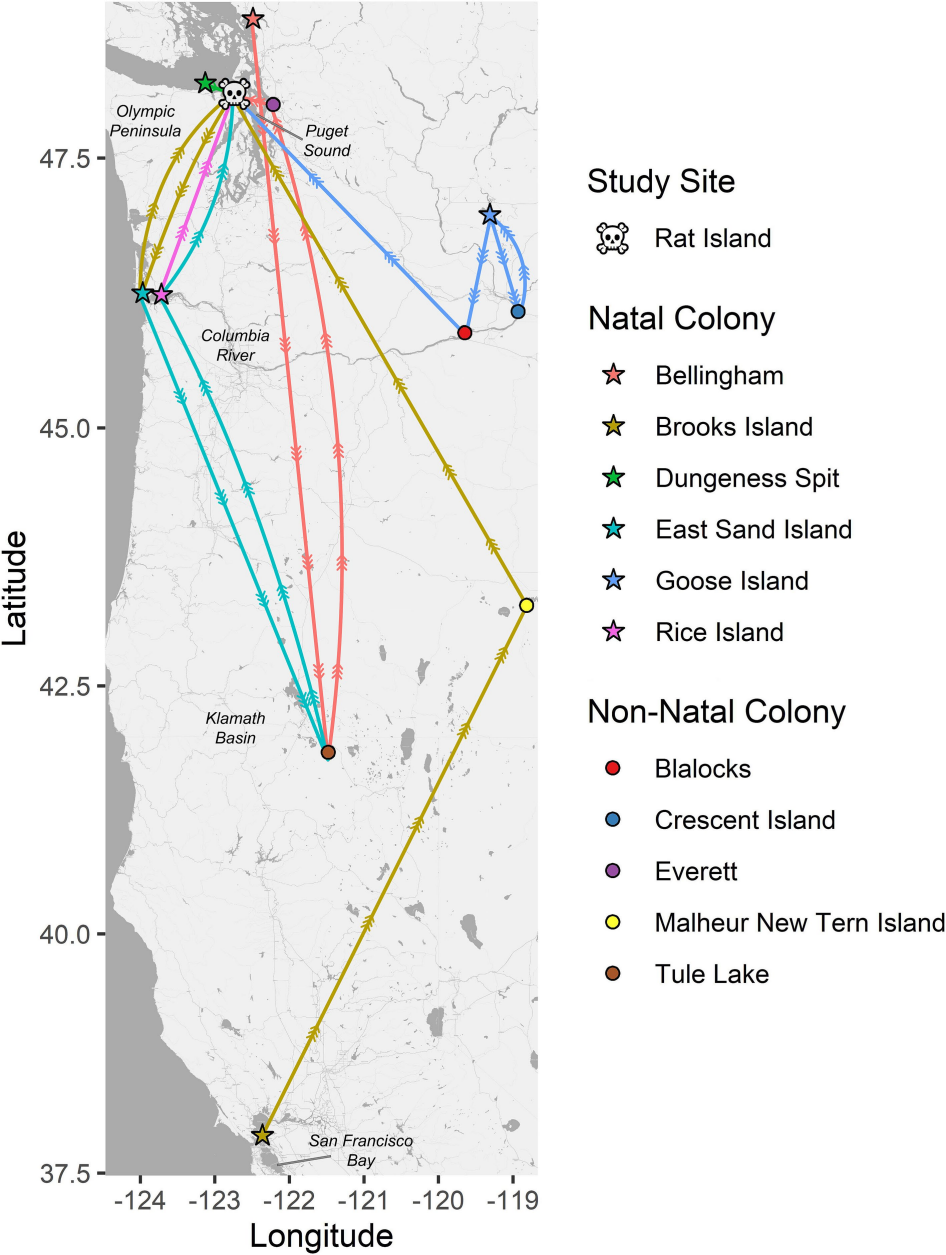
Pathways between natal colonies (stars) and resight locations for six color-banded Caspian terns (each represented by a unique color). All six of these birds died on Rat Island (skull and crossbones) in 2023 during the HPAI outbreak where their bands were recovered. The circles represent non-natal colonies where these birds were resighted. These six birds are representative of the resight pathways followed by the other 10 banded terns recovered on Rat Island and that were also banded on their natal colonies in Puget Sound, lower Columbia River, Columbia Basin (Goose Island area) and San Francisco Bay. These movement patterns have implications for the spread of diseases by terns and suggest that terns move both long and short distances among breeding colonies, they move regularly during their lifetimes, and that the most common pattern of movement, between the lower Columbia River and Puget Sound, is the pattern observed for the spread of H5N1 HPAI virus among terns in the region. Note that these movement patterns only represent movement among breeding colonies and not non-breeding movements to southern localities like Mexico or California and are likely biased by locations where there is a higher probability of detection and documentation such as where active research and observation is occurring.

Band data were recovered from 16 Caspian tern carcasses, including one that was captured/banded as an adult and all the others were banded as chicks. The birds were banded between 1997 and 2010 (13–26 years old) and were banded at relatively close locations 32–47 km from Rat Island (Dungeness Spit Washington, *n* = 1; Bellingham Washington, *n* = 1), locations 140 km to the southwest along the lower Columbia River (East Sand Island, n = 7; Rice Island, *n* = 1), 177 km to the east in the Columbia Basin (Goose Island, *n* = 1), and 1,132 km to the south in San Francisco Bay (Brooks Island, *n* = 1). Some of these birds were never resighted (*n* = 4) prior to their death. Most were resighted multiple times and one was resighted 142 times. All resights appear to be at breeding colonies. In Figure 2, we show representative movement patterns of six of these birds among colonies and regions based on their original banding location and their resight history. These do not represent annual movements but lifetime movements; a single colony location may represent multiple years breeding at that site before moving to the next site. The most common movement between colonies was between the lower Columbia River and Rat Island, which are the locations where we observed cases of HPAI in terns and matches the timing of mortality and the apparent movement of the disease based on our molecular results below. No movement data were available for seals.

### 3.4 Population estimates, mortality, and daily mortality rates

Only gulls, terns and seal carcasses were observed and collected on Rat Island, and we focus on those species here. Using the maximum number of animals counted from aerial photographs taken on July 17, 2023, we estimate there were 1,942 adult Caspian terns, 1,100 adult gulls and 130 harbor seals (7 of which were pups) on Rat Island (Table 1). For terns, we counted 1,045 nests in the photographs and the vast majority consisted of an adult sitting on the nest incubating eggs (Table 1). If we assume a pair associated with each nest, there were 2,090 breeding adults, which is a difference of only 148 terns from our maximum count of adults.

**Table 1:** Maximum number of live adults, pups (harbor seals only), and nests (terns only) on Rat Island at the time of the outbreak as determined by aerial photographs taken on July 17, 2023.

Counts of live animals		
Species	Adults (non-pups)	Nests/Pups
Caspian terns	1942	1,045
Glaucous-winged gulls	1,100	
Harbor seals	123	7

Counts of dead animals		
Species	Adults	Young-of-Year/Pups
Caspian terns	1,101	520
Glaucous-winged gulls	27	298
Harbor seals	6	10

Total counts of dead animals collected at four time points during the active H5N1 HPAIV outbreak and two time points post-outbreak. For harbor seals, numbers include stranded animals from the immediate surrounding area (Fort Flagler and Indian Island).

Using either the maximum count of adult terns (11,942) or doubling the number of nests (2,090), we estimate that at least 53–56% of the adult Rat Island tern population died during the event. Though we base our population estimate on aerial photographs collected at the onset of the H5N1 outbreak, this may be an underestimation because adult Caspian terns could have been away from the colony foraging at the time of the flight. It is also likely that some terns may have died elsewhere or that carcasses could have washed away with the tides and not been counted. In contrast to the Caspian terns, we estimate that only 2–3% of the adult gull population died. We do not know how many of the eggs from tern nests hatched (many eggs associated with tern nests did not hatch, presumably as the result of adults that died or became ill during the incubation period) and therefore we cannot estimate the proportion of the chicks lost. Nearly all of the gull nests had hatched just prior to the outbreak, and we could not get estimate gull nests or chicks because they were concealed in vegetation. Both gull and tern young of year died at high rates (Table 1). In addition to the mortality documented on Rat Island (Table 1), another 78 terns died in Everett, Bellingham, and Tacoma, Washington and 350 terns died along the lower Columbia River, primarily on Rice Island (*n* = 201) but also in Astoria and East Sand Island, Oregon. In total, 1,529 dead terns were found throughout western Oregon and Washington localities in July and August (Figure 1). We emphasize that these are minimal counts because many carcasses could have been washed out into the Salish Sea or gone undetected, especially along the lower Columbia River. For each of these locations, only a small subset of the dead or moribund birds were sampled for diagnostic testing but all carcasses tested were positive for H5N1 HPAIV (Supplementary Table S1).

**Figure 3:**
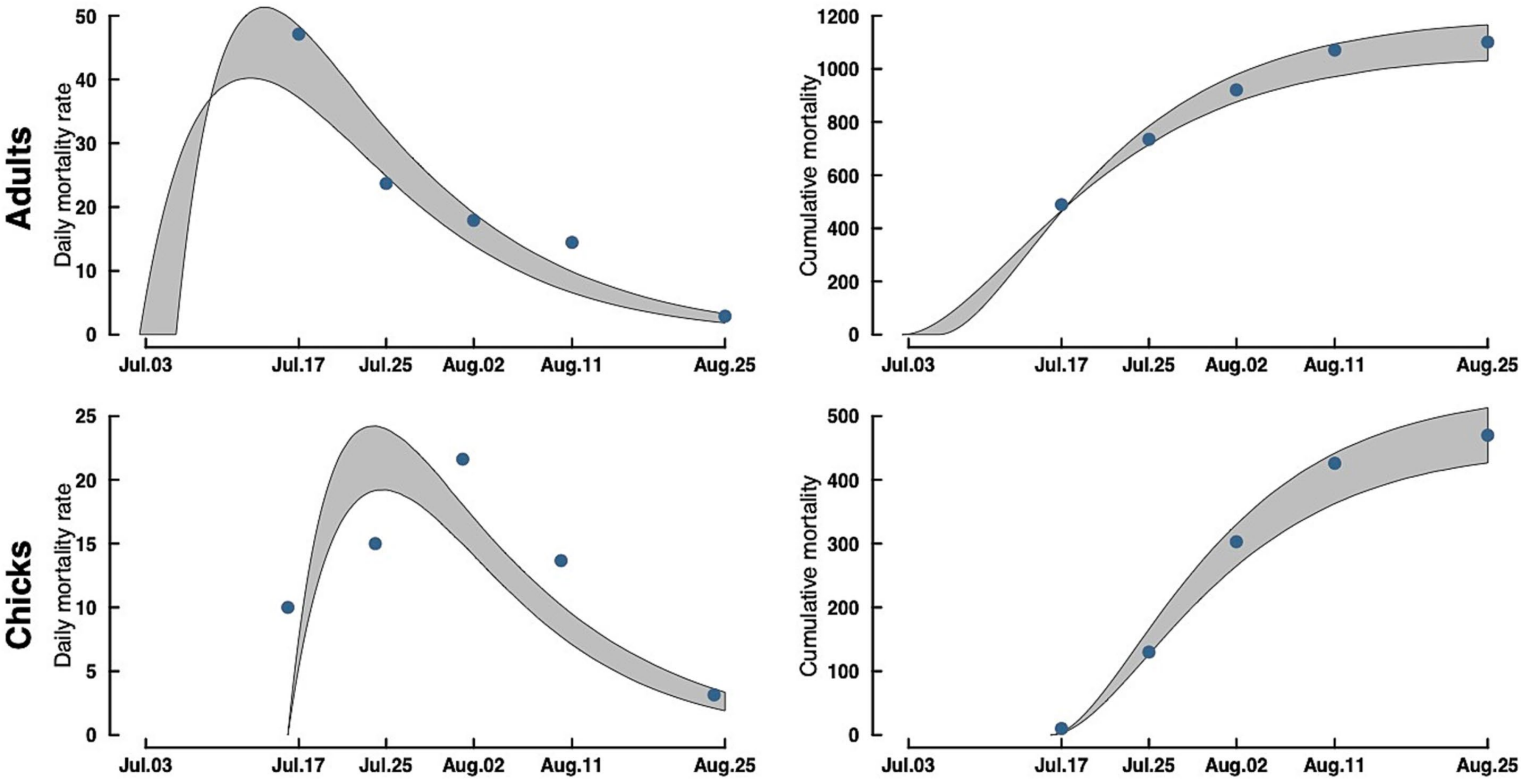
Adult (top) and chick (bottom) Caspian tern daily mortality rates (left) and cumulative mortality (right) during the 2023 Rat Island H5N1 HPAI virus event. Gray bands are a 95% CrI around the estimate. Dots in the accumulation curves represent the observed cumulative removals from the Island. The dots in the mortality rate curves are the actual counts of carcasses removed rescaled to daily mortalities (observed counts/ave. time difference between collections).

Our model of daily mortality for Rat Island suggests that adult terns started dying around July 3, 2023, and the outbreak lasted through the end of August 2023, with a peak in mortality around July 15, with approximately 50 bird deaths per day (Figure 3). For tern chicks, mortality started around July 17 (coincident with the first hatching nests) and lasted until the end of August, with the peak in chick mortality occurring around July 22 with approximately 24 deaths per day. Because hatching continued over several weeks, patterns of mortality in chicks differed from that of adults.

While it is possible not all harbor seal mortalities were recovered, given the surveillance efforts on Rat Island and adjacent Fort Flagler, we believe we found most harbor seal carcasses (*n* = 16), which indicates approximately 12% mortality of the harbor seal associated with Rat Island, based on the one count available (*n* = 130). Since we do not know how many pups were born on Rat Island during this outbreak, we cannot speak to the impacts of H5N1 on adults relative to juvenile harbor seals.

### 3.5 Harbor seal histology and immunohistochemistry

**Figure 4:**
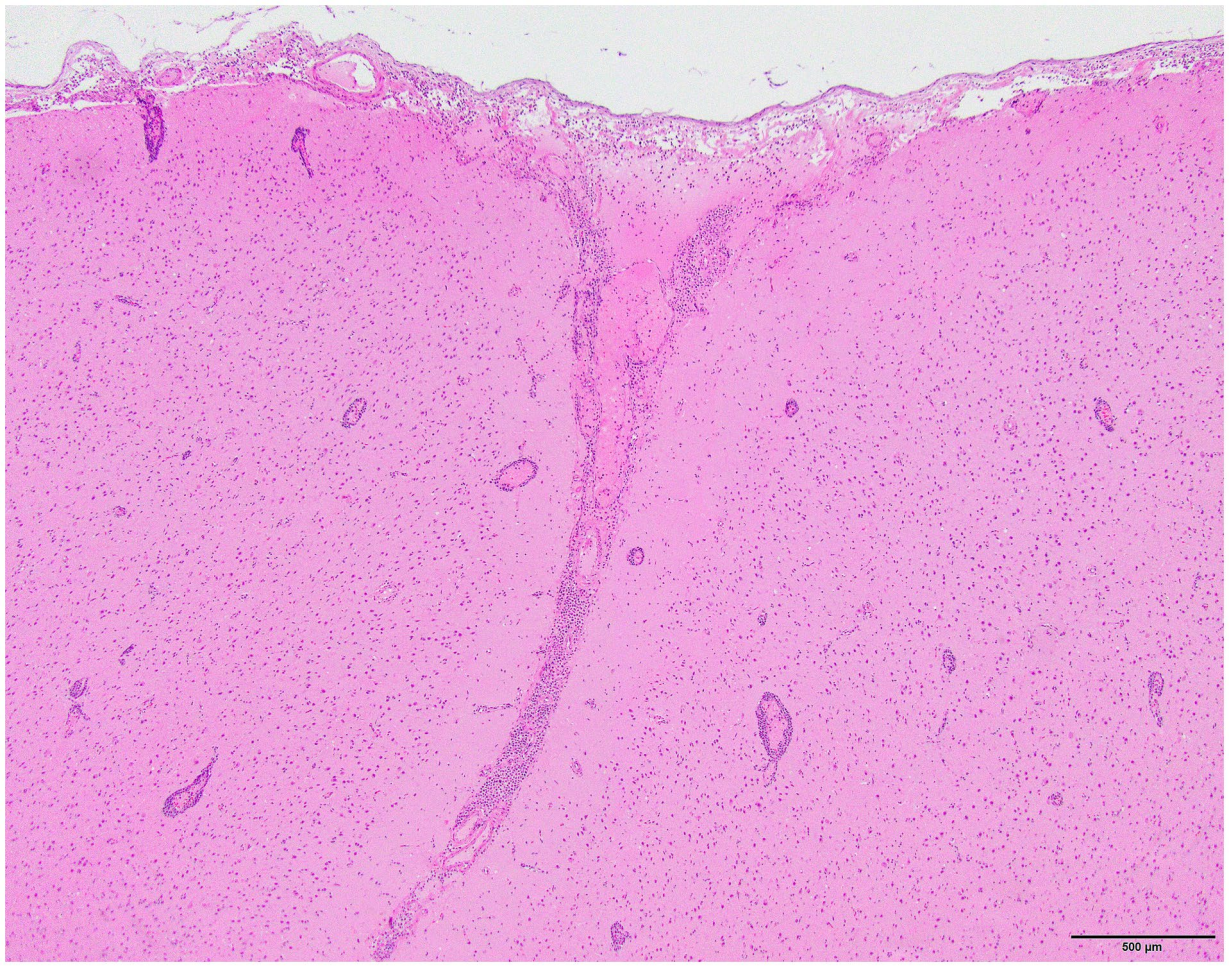
Harbor seal cerebrum exhibiting meningoencephalitis (H&E).

**Figure 5:**
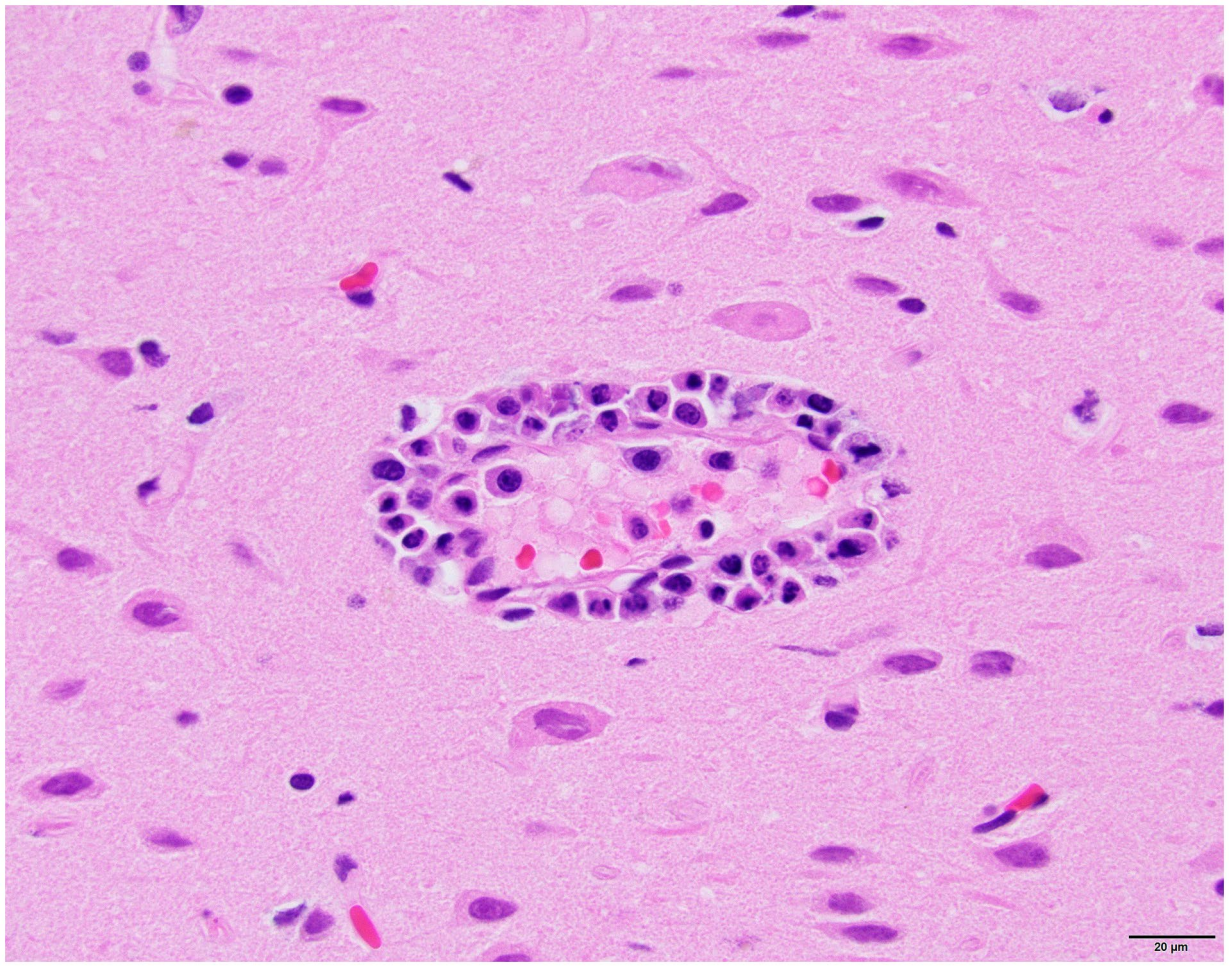
Harbor seal cerebrum. Cuffing in Virchow-Robin space, largely lymphoplasmacytic, associated with adjacent neuronal necrosis (H&E, 600x).

**Figure 6:**
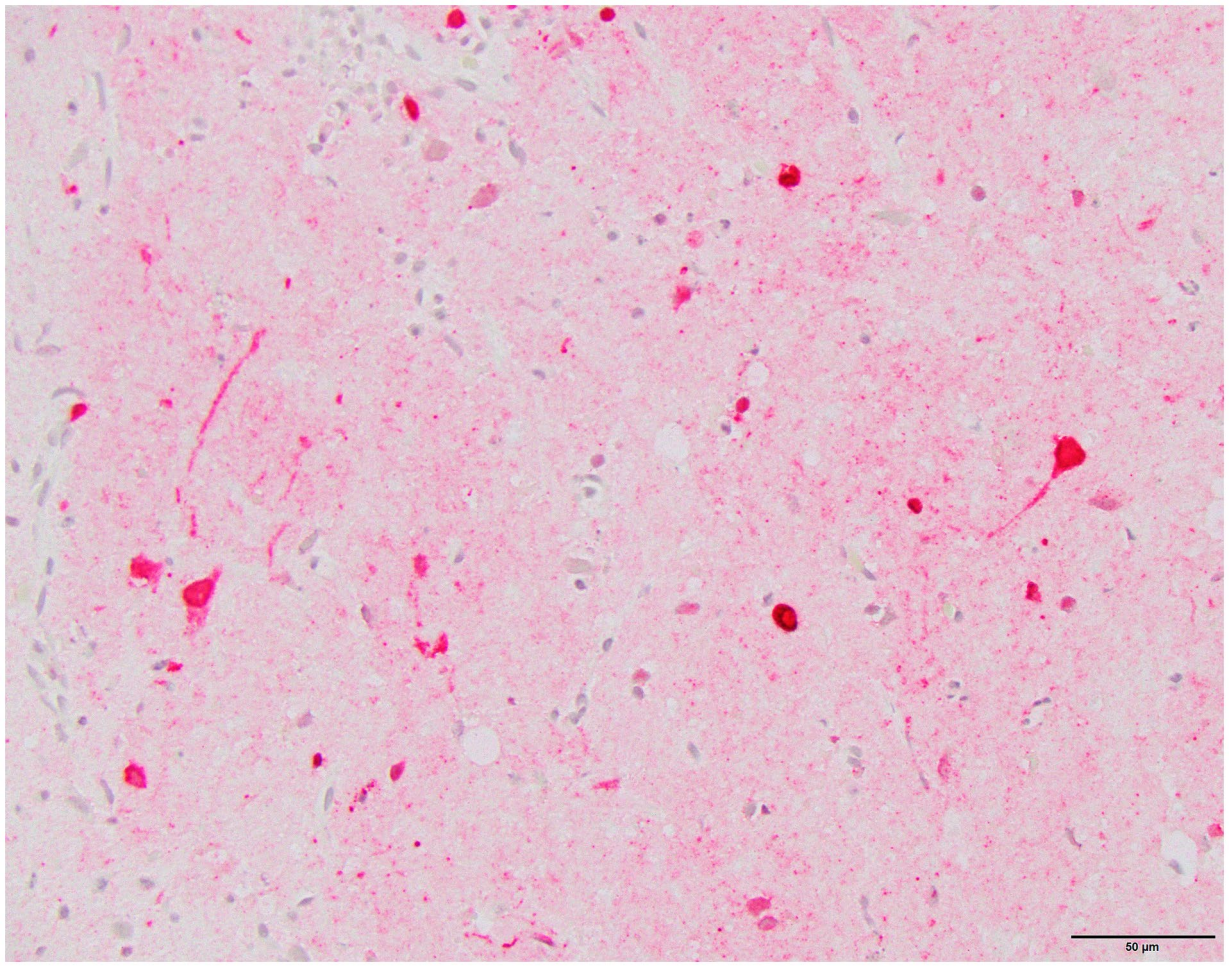
Harbor seal cerebrum. Immunoreactivity to influenza A nucleoprotein in neurons and glial cells (Immunohistochemistry, 400x).

Histology was performed on various tissues from all harbor seals confirmed to be positive for H5N1 HPAIV by RT-qPCR (Supplementary Table S1). Histologic findings included meningeoencephalitis, neuronal necrosis and perivascular cuffing, myocarditis, non-suppurative inflammation in the brain and heart, pulmonary edema and hemorrhage (Supplementary Table S1). For the cases with a meningeoencephalitis, all exhibited locally extensive or widespread neuronal necrosis and marked meningoencephalitis of the cerebrum and cerebellum (Figure 4), with a mixed leukocyte population of predominantly lymphocytes and plasma cells, as well as macrophages and rare neutrophils along with proteinaceous edema expanding the leptomeninges and Virchow-Robin space (Figure 5). Rarely, leukocytes extended into the neuroparenchyma. Immunohistochemical staining exhibited prominent immunoreactivity to the influenza A nucleoprotein in neuronal cell bodies and processes, as well as glial cells (Figure 6).

### 3.6 RT-qPCR for IAV detection

Twelve of 13 birds and five of 15 harbor seals screened for IAV via RT-qPCR ultimately tested positive for H5N1 HPAIV. Early in the outbreak, only nasopharyngeal swabs were tested from seals. Approximately 1 month into the outbreak, in mid-August, the marine mammal stranding network highlighted the relative increase in harbor seal mortalities in the immediate geographic area around Rat Island. Given this apparent increase, lung and brain tissue were tested as well as nasopharyngeal swabs from three seals that stranded on August 18 and August 25, 2023. Of these three, the nasopharyngeal swab from only one seal was positive for H5N1, while the tissues (lung and/or brain) from all three were positive for H5N1 HPAIV. Given these findings, archived tissues were submitted from the previously dead stranded cases for which tissues had been saved (Supplementary Table S1). Only two of the five harbor seals determined to be positive for H5N1 HPAIV had detectable concentrations of the virus on nasopharyngeal swabs.

### 3.7 Molecular epidemiology of H5N1 2.3.4.4b on Rat Island

**Figure 7:**
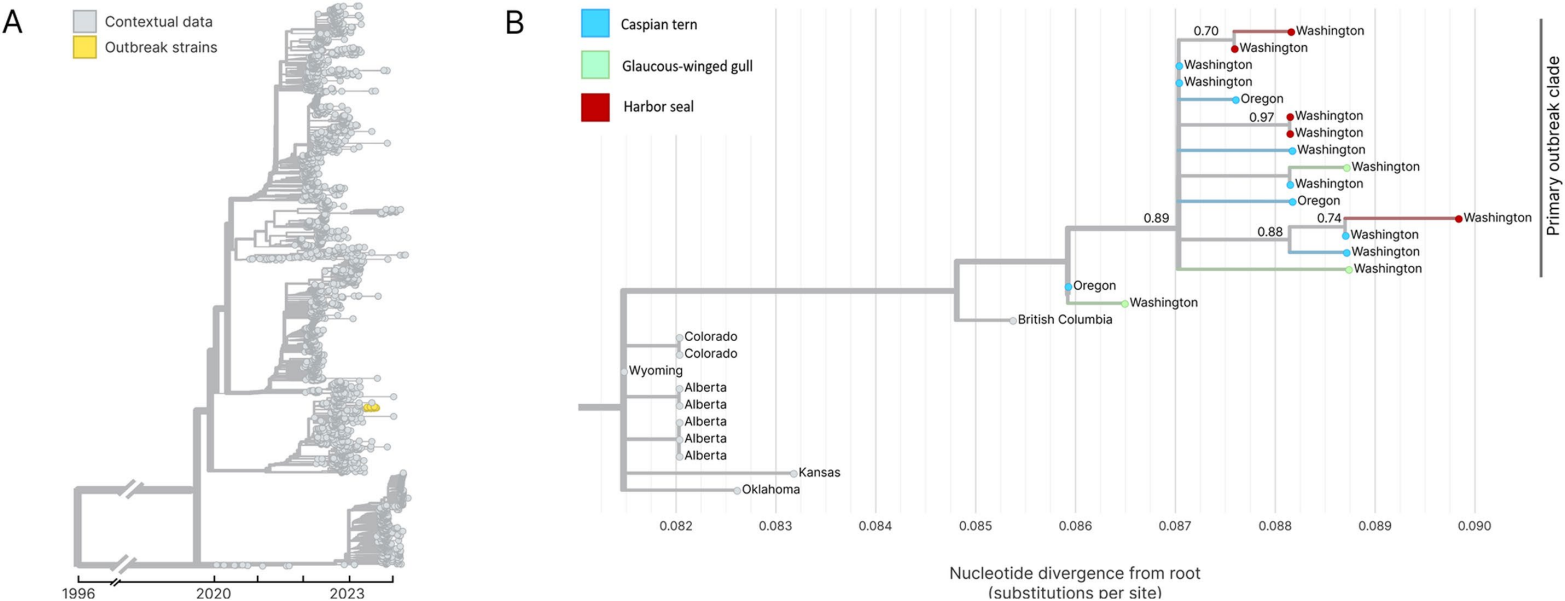
**(A)** Temporally resolved H5N1 HA segment phylogenetic with contextual data. Outbreak viruses are shown colored in yellow, contextual data is colored in grey. **(B)** Maximum likelihood genetic divergence tree of the H5N1 HA outbreak clade. Outbreak clade viruses of interest (colored yellow in panel A) are colored by host animal; light blue indicates a Caspian tern, green indicates Glaucous-winged gull and red indicates harbor seal. Geographic location where the virus was sampled from are annotated at the tips. Bootstrap support values are indicated at key nodes.

Whole genome sequencing of H5N1 HPAIV-positive diagnostic specimens yielded 17 complete IAV genome sequences from multiple animal species (12 bird; 5 seal) infected during the outbreak event and sampled between June 15 and August 31, 2023. These data include five sequences sampled from five unique harbor seals, nine sequences from Caspian terns sampled from three different geographic locations along the Puget Sound, and three sequences from infected gulls. Phylogenetic inference performed using HA segment sequences show that all sequences sampled during the outbreak event descend from a single common ancestor, indicating that this outbreak resulted from a single introduction event (Figures 7A,B). Fifteen of the sequences group together in a monophyletic clade with strong bootstrap support, which we refer to as the primary outbreak clade (Figure 7B). Two samples, one sampled from a gull in Oregon and one from a Caspian tern sampled during the outbreak even in Washington, outgroup the primary outbreak clade, though all of these sequences do still share a single common ancestor. The sequence sampled from the Oregon gull is the most basal sequence amongst the 17 sequences explored here and has an identical HA sequence to the inferred common ancestor of all 17 sequences. This finding is consistent with observed patterns of H5N1 detection in terns where mortalities were first observed in Oregon in June and then later in Washington in July, suggesting the Oregon transmission likely preceding the start of the outbreak in Washington. This timing is consistent with when outbreaks were observed to begin in these different regions, where dead birds were first observed in June in Oregon along the lower Columbia River and then in the Puget Sound Region of Washington in July and August.

Within the primary outbreak clade, we observe that the five sequences sampled from unique harbor seals cluster into three separate clades with bootstrap support of 70% or higher (Figure 7B). This is consistent with three separate virus introduction events from gulls or terns into harbor seals. Two of the clades group two harbor seal sequences together, while one clade groups sequences from a single harbor seal sequence and a single Caspian tern sequence. In one of the harbor seal clades, both harbor seal samples have identical HA gene sequences (Figure 7B). To further explore these spillover events and attempt to differentiate bird-to-seal and seal-to-seal transmission, we performed phylogenetic analysis and ancestral host state reconstruction. Given that the primary outbreak clade sequences are collected over a short time window, during an acute outbreak event, and had greater than 99% nucleotide identity across all genome segments, we did not expect reassortment to impact phylogenetic analysis of the primary outbreak clade sequences. For this analysis we therefore used the concatenated whole genome sequences.

**Figure 8:**
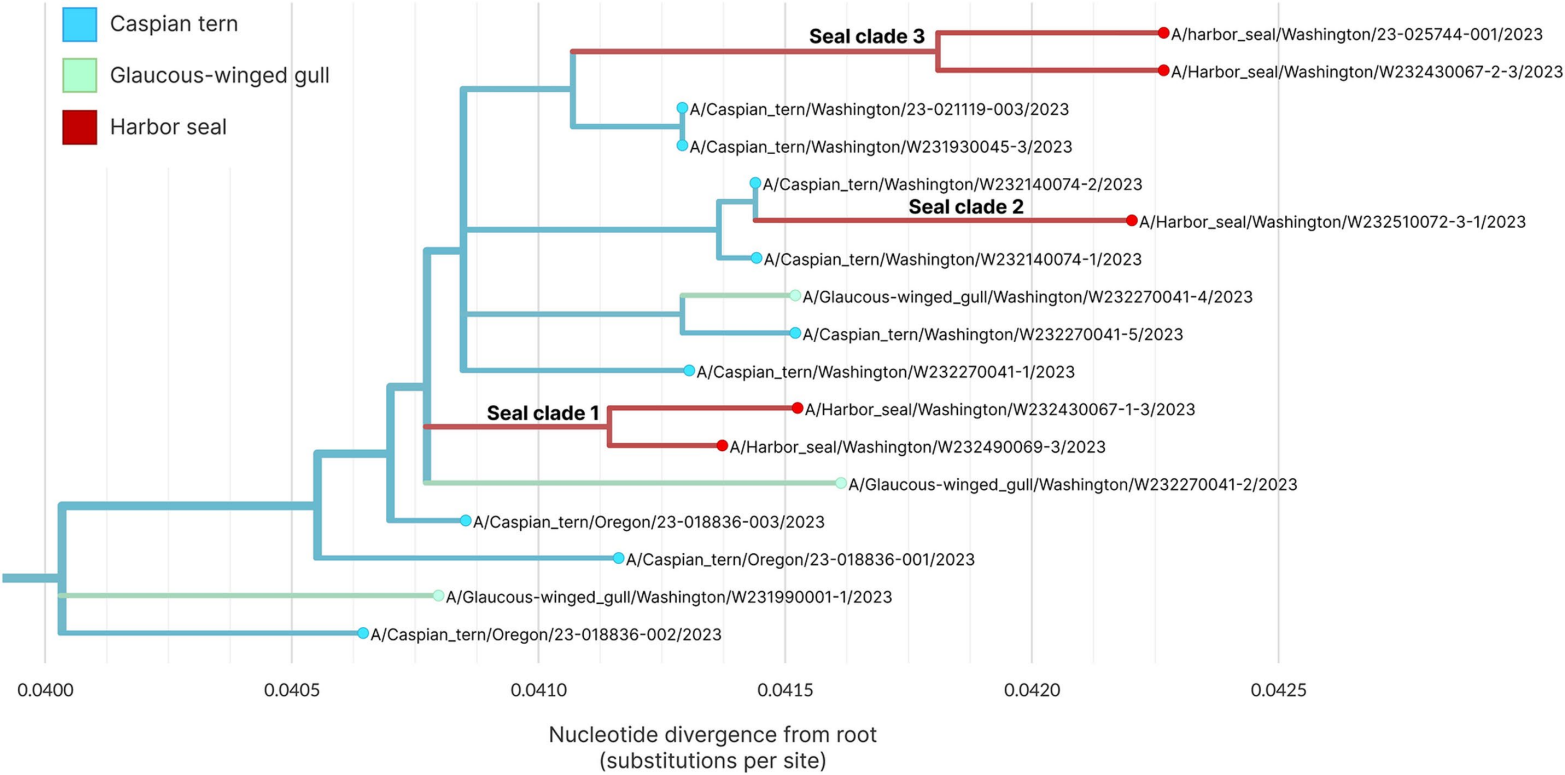
Maximum likelihood genetic divergence tree of the H5N1 whole genome outbreak clade. Tips and inferred internal node host state are colored by animal: light blue indicates a Caspian tern, green indicates Glaucous-winged gull and red indicates harbor seal. The three seal introductions are labeled.

Figure 8 shows the whole genome phylogenetic tree for the primary outbreak clade, with harbor seal clades indicated. Though use of the whole genome provides higher resolution to observe genetic distance resulting in different branch lengths between samples, the topology of the whole genome tree is consistent with the HA tree. As before, the data support at least three introductions of H5N1 HPAIV from terns into harbor seals. Harbor seal clades one and three (Figure 8) each group together two harbor seal sequences that descend from the same common ancestor and are more closely related to one another than to tern/gull viruses. The four nucleotide mutations defining Seal Clade 1 yielded no amino acid changes. Seal Clade 2 had one amino acid mutation in PB2 (E192K), which is a known, low-frequency mammalian pathogenicity-associated mutation that increases polymerase activity in mammalian hosts (43). Seal Clade 3 viruses shared three amino acid mutations (M646V in PB1, V11A in HA, and A30S in M2).

## 4 Discussion

During the H5N1 HPAIV clade 2.3.4.4b epizootic on Rat Island, Washington in summer of 2023, there were clear epidemiological patterns for infected host species, with host age class (adult vs. young of year) being an apparent key factor for mortality differences between terns and gulls. For both gulls and terns, conspecifics appeared to be in close contact and at relatively high densities. However, while both adult and young-of-year terns suffered very high mortality, only the gull young-of-year were similarly impacted by the H5N1 HPAIV. This result in adult mortality between species suggests differences in susceptibility and indicates that terns and gulls should not be viewed as having similar responses to IAV, especially H5N1 HPAIV.

Both gulls and Caspian terns are relatively long-lived species (several of the terns observed in this study were older than 20 years), which would provide ample opportunity for exposure to IAVs. It is well documented that many gull species are exposed to a wide diversity of LPAIV subtypes and adults yield a high seroprevalence when surveyed (44–46). This high level of LPAIV exposure may result in immunity and affords protection from H5N1 HPAIV infection or disease. While there is extensive data on IAVs infection in gulls, there are few corresponding studies for terns, especially Caspian terns. Interestingly, one study did find comparatively high seroprevalence in brown and lesser noddies, both terns in the family Laridae (47). This highlights the need for more surveillance and research in gulls and terns to better define the complex topic of IAV immunity in this group of birds, and complicates the interpretation of our results in the absence of serological data. Regardless, the pattern of mortality in adult and juvenile Caspian terns associated with the H5N1 HPAIV outbreak on Rat Island compared to gulls suggests that the Caspian terns may lack protective immunity to H5N1 HPAIV and all life stages are thus more susceptible to fatal infection. Understanding immune responses may not only help predict which species are most susceptible in the face of the ongoing H5N1 HPAIV wild bird panzootic, but also predict future temporal trends as the virus is maintained on land and seascapes.

In addition to prior exposure and immune status, H5N1 HPAIV disease characteristics may be influenced by viral dose and route of exposure. For example, the nesting ecology of Caspian terns is rather distinct compared to Glaucous-winged gulls. On Rat Island and elsewhere, the terns make nests, or scrapes, in a small area with the individual scrapes being extremely close to one another (35). In contrast, the gulls tend to nest on the margins of the island just above the rocky, debris filled high tide line. The gull nests are dispersed and further apart than terns. Given that H5N1 HPAIV can transmit directly or indirectly (through the environment) between birds, the proximity of the tern scrapes may have enhanced viral transmission in the nesting colony. Regardless, given that both the adult and juvenile gulls were scavenging moribund and dead Caspian terns, viral exposure and transmission in the gulls should have also been extensive. Ingesting infected tissues has experimentally been documented as a method of transmission for H5N1 HPAV in gulls (19). On several occasions, we observed gull chicks and adults scavenging/foraging on both dead and moribund terns, suggesting considerable exposure. The difference in mortality rates in adult Caspian terns compared to adult gulls highlights critical gaps in our understanding of host and viral dynamics and interactions that are key variables in driving disease and spread of H5N1 HPAIV across the global landscape.

Like many seabirds, Caspian terns have a relatively low annual reproductive output and relatively long-life expectancy (48). Based on information obtained from banded Caspian terns found dead on Rat Island, the age of the nesting adults ranged from 13 to 26 years. We estimate that at least 53–56% of the breeding adult tern population on Rat Island died during the H5N1 HPAIV event in 2023. While there were concurrent HPAIV-associated mortalities in other Caspian tern breeding colonies along the Pacific Flyway, tern carcasses were not systematically collected or counted, so the overall impact remains unknown. When we combined the number of dead breeding adult terns at Rat Island (*n* = 1,101) with other areas in Washington (*n* = 78) and those collected from Oregon (*n* = 350), the total observed mortality of Caspian terns in 2023 associated with H5N1 HPAI virus was 1,529. The 2021 flyway posteriori population estimate is 10,862 (49). Consequently, the proportion of adult Caspian terns lost during this mortality event in the Pacific Flyway was 10–14%. This is likely a minimum count of the 2023 tern mortality in Oregon and Washington due to the lack of a complete count of dead terns at other breeding colonies. This disease related mortality occurred when the population was already rapidly declining. A census-based minimum population estimate in 2021 indicates that the Pacific Flyway breeding population of Caspian terns declined by 54% since 2008, with most of this decline occurring between 2015 and 2021 (49). Our results indicate the population level impacts of these types of events and how they can compound population losses due to other factors.

The five harbor seals with H5N1 HPAIV virus clade 2.3.4.4b infection are the first documented cases of HPAIV in a marine mammal in the Northeast Pacific. Findings in the seals were similar to H5N1 HPAIV outbreaks in seals documented in 2022 in Quebec, Canada (50) and in Maine (51). Although there have been large mortality events associated with this virus in pinnipeds in South America (16), such extensive mortality was not observed in harbor seals in Washington or the other North American seal outbreaks. Given differences in apparent mortality rates between pinnipeds in South America and Washington, it is possible that, like the species-specific differences observed in susceptibility between the Caspian terns and gulls, such differences may also exist between species of marine mammals. Alternatively, the mechanism(s) of exposure may have differed between events as there is evidence that the H5N1 HPAIV strain affecting pinnipeds in South America may have possessed a greater ability to infect and/or transmit among mammals, compared to the virus infecting harbor seals in Washington (16). Further research is needed to better understand viral transmission dynamics and host susceptibility to H5N1 HPAIV in marine mammals.

The finding of brain positive samples when nasopharyngeal swabs were negative in seals highlights the importance of submitting multiple types of samples (e.g., nasopharyngeal swab, lung, brain) from wild mammals suspected to be infected/exposed to H5N1 HPAIV. Brain positive samples are consistent with observations in other wild terrestrial mammals (52), and these data highlight that H5N1 HPAI virus 2.3.4.4b has a strong tropism for the nervous system. This is supported in the histological findings from the harbor seals infected by H5N1 HPAIV that had widespread neuronal necrosis and a marked meningoencephalitis associated with H5N1 HPAI virus based on the immunohistochemical analyses. Interestingly, neurotrophic H5N1 HPAIV virus strains have not been reported in recent dairy cattle outbreaks, which have been detected in lung, mammary, and conjunctival samples (53, 54), highlighting that continued sampling of multiple organ systems are warranted in wild animals.

The H5N1 HPAIV sequences associated with the mortality event on Rat Island indicates very little genetic variation in the viral genome sequence during the active outbreak and mortality event (July–August 2023). This supports the host susceptibility differences we observed in the mortality data. More research is needed to better understand IAV exposure and immunological characteristics of Caspian terns relative to their gull counterparts on Rat Island and elsewhere.

Comparison of Washington and Oregon H5N1 HPAIV HA segments recovered from Oregon Caspian terns with those from Washington Caspian terns indicates that the virus was likely introduced to Washington from Oregon. This is also supported by the regular movement of banded birds between these regions and by the pattern of mortality, with the first virus detections and mortalities in terns observed in Oregon and then later in Washington. However, given the relatively sparse genomic sampling over the course of this event (*n* = 17), July–September 2023, this linkage may not be direct. The observed genomic pattern is also consistent with viral movement from Oregon to another, unsampled geographic location, with subsequent introduction to Washington. Under either scenario, genomic data support an outbreak event in Oregon preceding the Washington outbreak, with the Washington outbreak descending from the viral diversity circulating in Oregon.

Phylogenetic analysis of whole H5N1 HPAIV genomes sampled from avian hosts and harbor seals associated with the outbreak on Rat Island indicate that there were at least three unique avian-to-seal spillover events. This finding indicates that the mammalian outbreak was driven in part by multiple spillover events, though the reconstruction of two internal nodes as likely circulating in seals supports that some seal-to-seal transmission may have occurred as well. To better understand patterns of transmission both within and among species, more detailed sampling and viral sequencing throughout the outbreak would be necessary. In summary, we find strong evidence for multiple spillover events from terns/gulls into harbor seals on Rat Island. Local spillover from birds to mammals is supported by the fact that the seal event was limited in time and space, and no additional seal mortality events have occurred over the last 12 months. Finally, this local spillover from birds to mammals is consistent with the pattern observed in other outbreaks in North America (50, 51).

## 5 Conclusion

The global impact of H5N1 HPAIV clade 2.3.4.4b on wild birds and mammals is considerable based on raw mortality counts. However, few studies have accurately assessed the actual mortality and pattern of mortality across an entire outbreak or combined disease surveillance data with population monitoring (prior to the outbreak) to assess local colony and population impacts. Our investigation of the Rat Island outbreak employed comprehensive and multifaceted approaches that are uncommon in published studies of HPAI in free-ranging wildlife. In addition to the mortality event investigation, we also conducted population monitoring of Caspian terns at the onset of the outbreak, which allowed us to estimate total mortality. Using raw carcass counts collected nearly weekly during the outbreak, we were able to calculate population impacts of this outbreak on the terns. This was combined with diagnostic and genomic data confirming H5N1 HPAIV clade 2.3.4.4b infections in several hosts, including the first documentation of HPAI in a marine mammal in the Northeast Pacific. We document clear host susceptibility differences between breeding adult Caspian terns and gulls nesting at the same colony and in close proximity, with significant population level impacts for Caspian terns in the Pacific Flyway, yet very little impact to adult gulls. Finally, we found consistent patterns of the timing of H5N1 detection, the pattern of virus mutation, and the pattern of bird movement.

As comprehensive as our data are, we still lack a full understanding of these species’ immunological response(s) to IAV infections, especially H5N1 HPAIV, and how this affects susceptibility and disease outcomes in face of acute HPAI outbreaks and ongoing circulation of IAVs in the environment. This information gap and our findings highlight the importance of combining population monitoring, traditional and molecular epidemiological tools, and serologic surveillance for investigating wildlife morbidity and mortality events to better understand their impacts. We recommend expanding our wildlife health approach beyond mortality surveillance efforts (i.e., counting the dead) to also focus on population-level impacts and how environmental, behavioral, and immunological differences influence host susceptibility to disease, especially as H5N1 HPAI continues to devastate wildlife around the globe.

## Data Availability

The datasets presented in this study can be found in online repositories. The names of the repository/repositories and accession number(s) can be found in the article/Supplementary material.

## Footnotes

^1^gisaid.org
