# Supplementary material for: A comprehensive epidemiological approach documenting an outbreak of H5N1 highly pathogenic avian influenza virus clade 2.3.4.4b among gulls, terns, and harbor seals in the Northeastern Pacific
Source: Front Vet Sci. 2024 Nov 1;11:1483922. doi: 10.3389/fvets.2024.1483922 (PMC11565051; doi:10.3389/fvets.2024.1483922)
Supplement: Supplementary file 1 [file Data_Sheet_1.zip › Supplementary Figure S1.pdf]

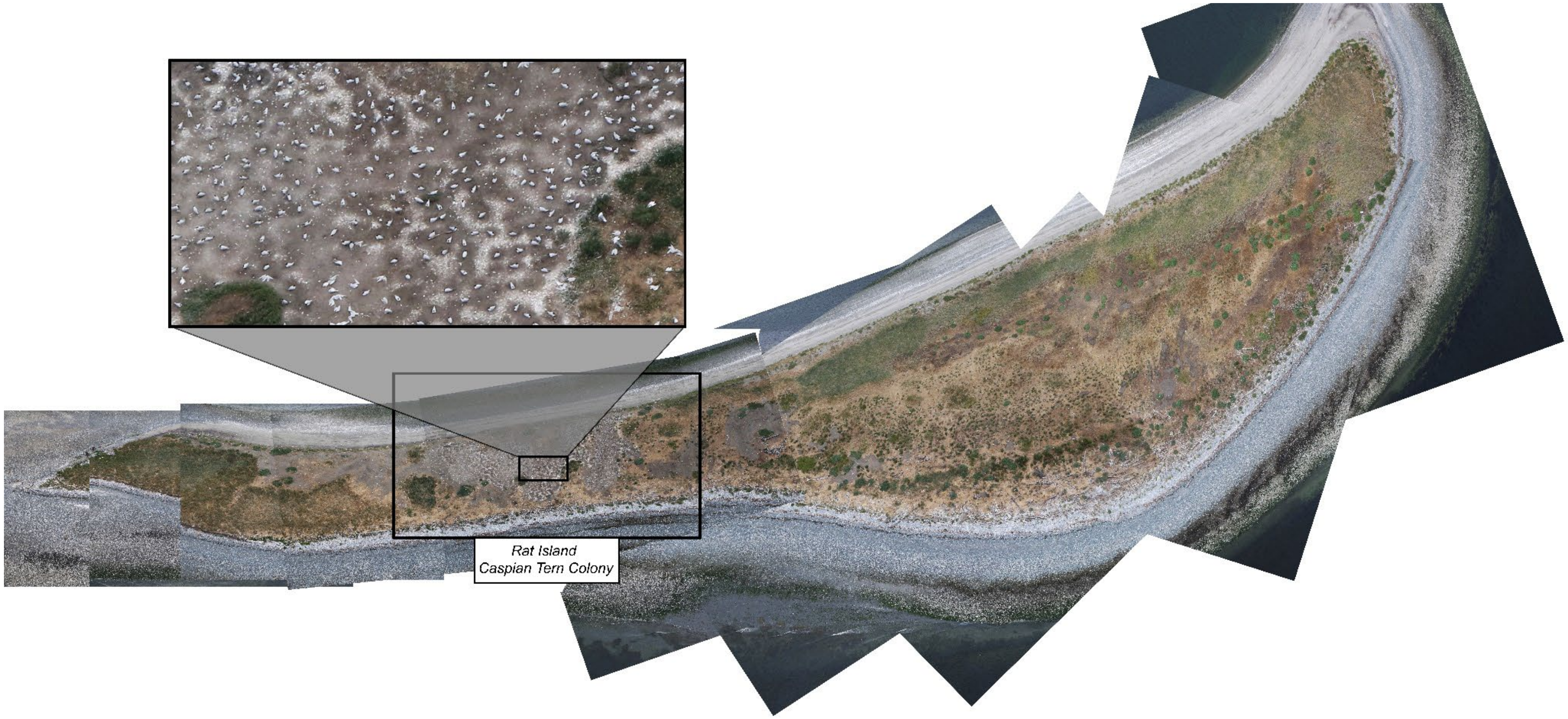

Supplemental Figure 1. Connected aerial photos taken from an airplane during a single pass over Rat Island on July 17, 2024. The Caspian terns (birds with black crowns and light grey backs) and their nests (grey colored depressions) are visible in the enlarged popped out portion of the image. A few Glaucous-winged-Western gulls hybrids (white heads with darker grey backs) and some tern carcasses are also visible in the enlargement.
